# Prospective organization of neonatal arm movements: A motor foundation of embodied agency, disrupted in premature birth

**DOI:** 10.1111/desc.12693

**Published:** 2018-06-19

**Authors:** Jonathan T. Delafield‐Butt, Yvonne Freer, Jon Perkins, David Skulina, Ben Schögler, David N. Lee

**Affiliations:** ^1^ Perception Movement Action Research Consortium The University of Edinburgh Edinburgh UK; ^2^ Laboratory for Innovation in Autism Faculty of Humanities and Social Sciences University of Strathclyde Glasgow UK; ^3^ Simpson Centre for Reproductive Health The Royal Infirmary of Edinburgh Edinburgh UK; ^4^ School of Physics The University of Edinburgh Edinburgh UK

**Keywords:** motor control, prospectivity, intentionality, neonate, embodiment, agency

## Abstract

Prospective motor control moves the body into the future, from where one is to where one wants to be. It is a hallmark of intentionality. But its origin in development is uncertain. In this study, we tested whether or not the arm movements of newborn infants were prospectively controlled. We measured the spatiotemporal organization of 480 full‐term neonatal arm movements and 384 arm movements of prematurely born infants at‐risk for neurodevelopmental disorder. We found 75% of healthy term‐birth neonatal movements and 68% of prematurely born infant movements conformed to the *τ*_*G*_‐coupling model of prospective sensorimotor control. Prospective coupling values were significantly reduced in the latter (*p* = .010, *r* = .087). In both cases prospectively controlled movements were tightly organized by fixed‐duration units with a base duration of 218 ms and additional temporal units of 145 ms. Yet distances remained constant. Thus, we demonstrate for the first time a precise prospective spatiotemporal organization of neonatal arm movements and demonstrate that at‐risk infants exhibit reduced sensorimotor control. Prospective motor control is a hallmark of primary sensorimotor intentionality and gives a strong embodied foundation to conscious motor agency.

**RESEARCH HIGHLIGHTS**

- Neonatal infant arm movements are prospectively organized.
- Infants born premature and at‐risk for neurodevelopmental disorder exhibit reduced prospective organization of arm movements.
- Prospective movement in neonates is organized by fixed duration, variable distance units.
- Prospective control of movement is an early foundation of embodied agency.

## 1 INTRODUCTION

Actions carry one into the future. Prospectively controlled actions bring one from “where one is” to “where one wants to be”. Actions made with an anticipation of their future effect and with control to achieve a desired aim are the quintessential expression of the mind, achieving in physical effect that which is literally “in mind”. Nobel laureate Roger Sperry (1952) remarked, “the sole product of brain function is motor coordination” (p 297). In this paper we probe the origins of prospective control of movement, movement with an eye to the future.

Neuromotor control of action is thought to employ a prospective view to achieve movements of the body that bring the person usefully forward in time (von Hofsten, 1993, 2004). Prospective movements are typically self‐generated and always organized by their future effect. But when in development this prospectivity first arises is not well understood. In this study, we asked whether or not the arm movements of newborn infants are enacted by prospective neuromotor control, and if so, whether or not this system might be compromised by neurodevelopmental challenge in the case of premature birth. This first fundamental question has implications for understanding the experiences of very young infants, and the ontogenetic origins of a basic, primary motor agency (Delafield‐Butt & Gangopadhyay, 2013). The second addresses the utility of a quantified measure of prospective control of movement in clinical assessment, and its possible developmental repercussions. We explore each of these in more detail below. Altogether, this work has implications for understanding the development embodied psychological experience by giving precise definition to the emergence of motor agency and intentionality in early life.

### 1.1 Early development of agency

The origins of neuromotor control appear in the first writhing movements of the late stage embryo from which they are “born”. Over foetogenesis they become differentiated, defined and organized, becoming well established as organised action by mid‐gestation.

Spontaneous self‐generated movements are seen to occur *in utero* as early as 7 weeks gestational age (Lüchinger, Hadders‐Algra, van Kan, & de Vries, 2008). At this first stage, movements of the arms are associated with writhing movements of the trunk and whole body (de Vries, Visser, & Prechtl, 1982; Prechtl, 1986). Soon after this earliest onset of movement, at 8 to 10 weeks gestational age, foetal arm movements can become directed to parts of the body, especially to the face and head (Piontelli, 2010). Over weeks 10 to 14, foetal arm movements become increasingly differentiated into individual, isolate actions. And their form of movement becomes increasingly organized by their end effect, generating sensory contingencies on arrival at their goal.

At this early age, developing neural systems for proprioceptive feedback gives a regulatory feedback loop over the course of the action made via the spinal cord and developing central nervous system (Butterworth, 1986). Interestingly, autostimulation appears an important influence on the form and choice of action. Ultrasound observations show that foetuses may explore their forehead region adjacent to the anterior fontanelle, at the border of trigeminal (fifth cranial) nervous innervation (Piontelli, 2010). As the nerve innervates the forehead and spreads, so the foetus’ hand and finger movements—rich with sensory innervation themselves—follow this border, exploring through touch the edge of innervation. The boundary of innervation gives a two‐sided response to touch. On the innervated side, populated with sensory nerves along the surface of the head, the action of a touch gives both proprioceptive and autostimulatory feedback; on the uninnervated side, without sensory fibres, the same action gives the same proprioceptive and tactile feedback in the fingers, but without an accompanying autostimulation on the head. The foetus appears to be exploring the boundary of her sense of body‐self through proprioceptive and autostimulatory feedback. Later in foetal development, greater explorations of self and environment can be observed with differential actions of the arms to touch the eyes, the mouth, the uterine wall, and so on, and individual behavioural characteristics begin to appear, such as a propensity to fondle the umbilical cord, scratch at the placenta, or, indeed, make twin‐directed movements (Jakobovits, 2009; Piontelli, 1992, 2002; Reissland, Francis, Aydin, Mason, & Schaal, 2014).

At 14 weeks gestational age, a quantified kinematic analysis of foetal arm movements in twin pregnancies revealed a prospective organization of action structured by its final contact with a particular object, its extrinsic “goal” (Castiello et al., 2010). Special twin‐directed movements were evident. By 18 weeks gestational age these had differentiated significantly from those actions used to touch inanimate objects in the environment (umbilical cord, uterine wall), but remained similar in form to those made toward one's own eye—indicative of a sensitive early social awareness. In a comparable study of singleton pregnancies, kinematic studies confirm that motor planning was operative by 22 weeks gestational age (Zoia et al., 2007). These foetal studies demonstrate a prospective awareness of the end effect of an action on its environment, detected by the distance and touch receptors of the eyes, ears and fingers, its kinematic forms made in the present moment were structured by knowledge of its future sensory effect.

Prospective awareness is also evident in anticipatory movements of a target part of the body during self‐directed arm actions. For example, 4D ultrasonography shows the mouth of the foetus to open in anticipation of being touched, made during a mouth‐directed movement but before actual contact (Myowa‐Yamakoshi & Takeshita, 2006; Reissland et al., 2014). Again, an awareness of a self‐generated future made through movement and evident in its sensory contingencies was apparent. Altogether, these studies suggest that the development of prospective, anticipatory awareness in motor control emerges at the beginning of foetal life at the end of the first and beginning of the second trimester. This is an emerging knowledge of endogenously generated action outcomes contingent on distance and touch receptors to sense the effect of an action on the external world.

This basic capacity to know the outcome of a self‐generated action is a core feature of embodied psychological experience and the beginning of what Piaget identified as “sensorimotor intelligence” (Piaget, 1953, 1954). This is an “action‐response” mode of cognition predicated on agent action, rather than a passive “stimulus‐response” one that does not account for spontaneous movement. The former is ubiquitous and dominant in infant and child life, but it is the latter that is typically the focus of experimental study—an imbalance in current awareness in cognitive science that we seek to redress.

The recent *in utero* behavioural data on foetal action development are at odds with conventional wisdom that neonatal arm movements are simple reflexes without a psychological dimension. There are indeed reflexes that are actions produced under particular sensory stimulation, such as the induction of rapid arm abduction in sudden free‐fall (the Moro reflex), but these are unusual and particular stereotyped reactions to sudden environmental changes; they do not constitute the typical, ever‐active arm movements made by newborns regularly when left still or when in regular communication with an adult. These latter, spontaneous arm movements are self‐generated by the infant, they are endogenous, they come from within. And they can be useful in specialized clinical assessments of neuropsychological integrity as well as to become a reference point to assist maternal bonding through recognition of an infant's agency (Brazelton & Nugent, 1995; Einspieler & Prechtl, 2005; Spittle, Doyle, & Boyd, 2008). But these self‐generated “general movements” of infants are not well studied in psychology. There are only a very few studies that employ computational motor assessment methodologies. Nor have they been very well considered as an important feature for the early ontogenesis of children's agency (Nagy, 2011; Zeedyke, 1996). This is a shortcoming in our subject, because as the “sole product of brain function” arm movements in particular are amenable to very sensitive, very precise measurement at sample rates sufficient to assess neurophysiological function (500 Hz), and with less invasive and less costly technique than with, for example, brain imaging. This was our motivation for including a prematurely born infant population at‐risk for neurodevelopmental disorder.

Only a handful of researchers have characterized these actions with quantified precision. The first studies to do so employed optical motion capture technology to track neonatal arm movements (von Hofsten, 1991; von Hofsten & Rönnqvist, 1993). These studies found that the movements were structured by phases of acceleration and deceleration, termed “movement units”, that coincided with spans of movements along the longitudinal, dorso‐ventral, or lateral axes of the infant. Shifts in movement direction occurred more frequently at the ends of these movement units. Another study investigated newborn arm movement patterns from a dynamical systems perspective, using inertia analysis from accelerometers (Ohgi, Loo, Morita, & Mizuike, 2007). They also found that neonatal movement patterns were structured, this time along a five‐dimensional embedment. Other studies have performed examinations of the exploratory use of the hands (Jouen & Molina, 2005; Molina & Jouen, 2004), but research on newborn arm movements remains scarce. How the newborn controls arm movement to produce the kinds of structures observed above, and whether or not these are prospectively controlled, remains an open question.

It is clear from previous work that neonates develop prospective awareness of their action outcomes and they may control movement to achieve a particular sensory effect. For example, in an early reach‐to‐grasp task, Bower, Broughton, and Moore (1970) employed a visual illusion to demonstrate that infants anticipated contact with an object when they reached for it, and became distressed if contact—and its tactile sensory consequences in the fingers—was not made, because of the illusion. Similarly, a school of work has developed to demonstrate infants as young as 3 months lying supine may learn to perform a particular kicking pattern with the legs, when those legs are geared to an attractive mobile that only responds to that particular kicking pattern (Angulo‐Kinzler, 2001; Angulo‐Kinzler, Ulrich, & Thelen, 2002; Fagen & Rovee, 1976; Rovee‐Collier & Gekoski, 1979; Rovee‐Collier, Morrongiello, Aron, & Kupersmidt, 1978). Again, the model tested in these paradigms is that sensory consequences of a particular action are learned, then anticipated during an intended action that seeks to re‐create them. This model implies that the actions of the infants are first mechanical motor activity, their perceptual monitoring and reward can then be remembered to guide subsequent volitional action—the purported source of intentional agency. This is a notion that treats volitional agency as ontogenetically disembodied, requiring additional cognitive oversight to become acted through the body.

In this paper, we challenge the notion that the spontaneous, self‐generated general movements of the infant can be simply mechanical motor acts devoid of intentionality. Instead, we hypothesize that they might be perceptually and prospectively controlled within the nature of their psychomotor structure. In our model, each of the above qualifiers of control is important and substantiated. They must be *prospective* by necessity of lawful biomechanics (see below and see Bernstien, 1967), *perceptually* controlled toward that prospective end‐state, its conclusion or “goal”, over the course of the movement to ensure that it is reached parsimoniously and with useful effect (see Lee, Bootsma, Frost, Land, & Regan, 2009), and that this structure is intrinsic to the neuromotor system itself. Thus, we challenge the status quo model of pure physiological neuromotor control without the necessity for prospective guidance, with one that includes perceptual and prospective elements within the system, a strong embodied system of neuropsychomotor control. Such prospective control is not necessarily contingent on external sensory consequences, but may be structured within the integrated activity of the nervous system. Such psycho‐physical prospectivity is thought to give coherence of psychological purpose together with coherence of the forces across the body in movement, presenting a coherence of psycho‐motor activity important for learning and development (Delafield‐Butt & Gangopadhyay, 2013; Lee, 2005; Lee et al., 2009; von Hofsten, 1993, 2004).

### 1.2 Prospective control of movement—tau theory

Within perceptuomotor control theory, the biomechanics of human movement suggests that self‐generated arm movements must be prospectively controlled, because a movement generates forces and momenta that have to be balanced before they upset the flow of action of the body (Bernstein, 1967; Trevarthen, 1984). The course of a movement must be prospectively prescribed (von Hofsten, 1993; von Hofsten, 2004). We decided to make exact measurements of newborn arm movement using high‐precision motion capture to test the form of these actions against a mathematicopsychophysical theory of movement control that has at its core a postulated prospective information variable, the τ variable in general tau theory (GTT) (Lee, 1998; Lee, Craig, & Grealy, 1999; Lee et al., 2009).

We chose GTT because: (i) it is a mathematical model that predicts a form of action pattern if the action is under prospective control; (ii) it thereby enables a statistical means of testing neonatal arm movement patterns for prospective control and a quantified means of analysing them; and finally (iii) the theory has roots for explaining the dual psycho‐physical nature of motor behaviour that extend deeper into a natural science of psychological embodiment than do descriptive kinematic measures. The theory argues that animate movement is the physical aspect of a psychological phenomenon based on neuromotor activity on the one hand and perceptions, feelings, and intentions on the other (Reed, 1996). GTT purports to identify the information directly experienced and controlled in an action, whereas kinematic measures merely identify third party descriptors of the movement. GTT is a general theory of perception‐action motor control developed from the work of James J. Gibson (1966) and Nicolai Bernstein (1967). It advances their work to give a mathematical account of how intentional movement can be prospectively guided, and its conclusions are supported by experiments spanning a wide range of perceptually controlled animal and human skills (for reviews, see Lee, 2005; Lee et al., 2009).

**Figure 1:**
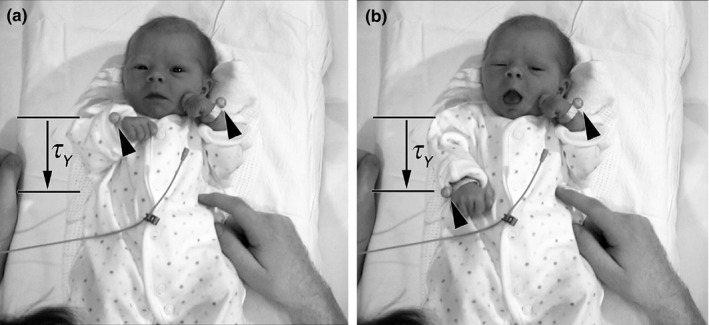
Example infant arm movement viewed from above. The infant lay supine on a padded table with a reflective marker attached to each wrist (arrowheads). The movements of the markers were recorded with a 4D motion tracking system at 500 Hz. In this example, our model predicts the longitudinal component of movement (parallel to the infant's vertebral axis) of the right arm, from near the shoulder (a) to a full extension to the waist (b) is controlled by coupling, τ_*Y*_ (the tau of the gap, *Y*, to the end of the movement) to an intrinsic tau guide, *τ*_G_. An example of the resultant velocity and tau coupling is given in Figure 2. The infant's parent sat at the foot of the table

No study has yet been conducted to test for an intrinsic prospective organization of neonatal arm movement motor control. We designed our study to determine whether or not infants’ arm movements are prospectively organized. Our hypothesis is that a newborn moving her hands from, for example, her shoulder to near her waist, which is a typical neonatal arm movement, necessarily involves prospective guidance (Figure 1). We reasoned that the baby's brain prescribes an ideal form for that movement, taking into account the dynamical constraints of the body. Our theory suggests that it does so by creating a temporal pattern of neural activity that can be coupled to the enacted action pattern to give the movement a prescribed form. On the perceptual side, the infant is afforded information of her arm's action pattern through her arm's proprioceptive apparatus and her visual proprioception of her arm (van der Meer, van der Weel, & Lee, 1995). Coupling her sensed action pattern to an intrinsic action pattern by regulating her muscles results in a prospective structure within the action of her body that adapts dynamically to its neurally prescribed form (Lee et al., 1999). This system of prospective sensorimotor control does not require knowledge of additional extrinsic sensory consequences, but is already structured by the neuromotor system. As such, we believe it to be a fundamental neuromotor structure that underpins more complex associations of stimuli received at the ends of actions, and that can be used to engage with objects and people in goal‐directed and expressive gesture (Schögler, Pepping, & Lee, 2008; Lee et al., 2009).

The mathematics of general tau theory provides an explanation of how an activity like moving the arms is intrinsically and perceptually guided. The theory is based on the prospective informational variable, τ_*Y*_(*t*), which equals at any time, *t*, the time‐to‐closure of a gap, *Y*(*t*), at the current rate of closure, $\dot{Y}\left(t\right)$. Thus, τ_*Y*_(*t*) equals $Y\left(t\right)/\dot{Y}\left(t\right)$. Only information about τ_*Y*_(*t*) is required to control the closure of a gap: information about the size, *Y*(*t*), and rate of closure, $\dot{Y}\left(t\right)$, of the gap is not needed to control its closure. This provides a very efficient system for the control of purposive movement with a low computational load on the nervous system. Furthermore, the *τ* of a gap is *directly* perceptible in any sensory modality, in contrast to spatial quantities such as the size and rate of closure of a gap, which are generally *not* directly perceptible (Lee, 1998).

**Figure 2:**
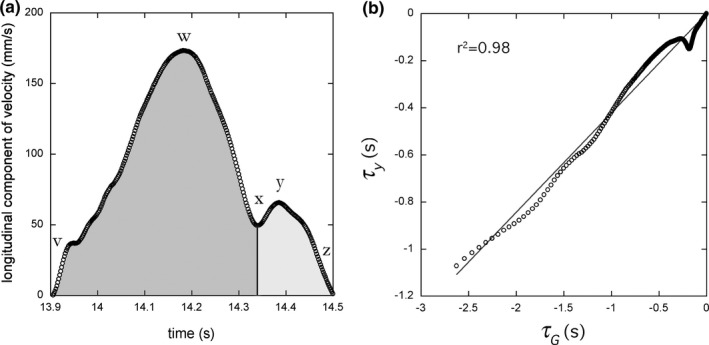
(a) The longitudinal component of velocity, $\dot{Y}\left(t\right)$, parallel to the infant's vertebral axis of a typical action unit (see Figure 1). In an action unit this component of velocity is continuously positive or negative. Within an action unit there may exist one or more *movement units*, defined as a phase of acceleration followed immediately by a phase of deceleration. In (a) the movement accelerates from zero velocity (at v) to a peak velocity (at w), then decelerates before accelerating again (at x) briefly, producing a second, smaller velocity peak (at y) and decelerating again to zero velocity (at z). Thus, this single action unit is composed of two movement units, one (dark grey) from v to x and another (light grey) from x to z. (b) The tau of this full action unit, *τ*_*Y*_(*t*), is coupled to the tau of its intrinsic guide, *τ*_*G*_(*t*, *T*_*G*_). In this example, the average coupling constant, *k*_*Y*,*G*_, given by the slope of the linear regression over the last 90% of the movement, was 0.33, so that, *τ*_*Y*_(*t*) = 0.33 *τ*_*G*_(*t*, *T*_*G*_). The proportion of variance accounted for by this *τ*_*G*_ ‐coupling equation was 0.98 (the r^2^ of the regression)

We reasoned that in spontaneous neonatal arm movements, tau information may be used to guide displacement of the arm up (postero‐anterior) and down (antero‐posterior) the neonate's vertebral (longitudinal) axis. Our hypothesis is that the infant picks up propriospecific information about the tau of the action gap between the current position of her arm and an end‐goal position, which lies within the range of the arm movement. She uses this information to regulate her muscles to keep the tau of her arm movement coupled, in constant proportion to a projective tau, τ_*G*_(*t*, *T*_*G*_), generated by patterned nervous activity. A sample movement is shown in Figure 1. Corresponding velocity and tau values calculated over the course of a movement are given in Figure 2.

We applied this model (Box 1) to test for prospective control by calculating the τ_*Y*_(*t*) of each arm movement and measuring how closely coupled, in constant proportion, it was to τ_*G*_(*t*, *T*_*G*_). Such τ_*G*_ *‐coupling* is evident in a range of adult human actions, including reaching (Lee et al., 1999), intercepting (Craig, Pepping, & Grealy, 2005), and golf putting (Craig, Delay, Grealy, & Lee, 2000). Importantly, the tau information variable employed in τ_*G*_ *‐coupling* is contingent on goal‐acquisition—it is a measure of the action‐gap, the gap to the future goal. Therefore, tau is prospective information by its mathematical definition. We reasoned that if the infants’ movements were significantly τ_*G*_ ‐coupled there would be reason to believe that these movements were prospectively controlled. And we reasoned that if infants were at‐risk for neurodevelopmental disorder, such as in the case of prematurely born infants, this coupling might be compromised. Identification of such a disturbance to movement in this population would have potential clinical importance for understanding the motor contribution to developmental psychopathologies, such as, for example, in the case of autism spectrum disorder where premature birth introduces greater prevalence (Limperopoulos et al., 2008).

Box 1 **Tau model of prospective motor control**

τ_*G*_(*t*, *T*_*G*_) equals the tau of a gap, *G(T)*, between the current level of neuro‐power (the rate of flow of electrochemical energy along neural axons) and a reference level. *G(t)* closes from rest at constant acceleration. The function, *τ*_*G*_(*t*, *T*_*G*_), is derived from Newton's equations of motion as

(1) $$\tau_{G}\left(t,T_{G}\right)=\frac{1}{2}\left(t-T_{G}^{2}/t\right)$$

where time, *t*, runs from zero to *T*_*G*_, the duration of closure of the gap, *G(T)*.

The model predicts that skilled, self‐timed closure from rest of an action gap, *Y(t)*, will follow the *τ*_*G*_ *‐coupling* equation:

(2) $$\tau_{Y}\left(t\right)=k_{Y,G}\tau_{G}\left(t,T_{G}\right)$$

where *k*_*Y*,*G*_ is a constant during the movement. Only when *k*_*Y, G*_ = 1 does the action gap, *Y(t)*, close with constant acceleration like *G(t)*. Otherwise, when 0 < *k*_*Y, G*_ <1, the action gap accelerates then decelerates, and the value of *k*_*Y*,*G*_, which is assumed set in the nervous system for each particular movement, controls the shapes of the velocity and acceleration‐deceleration profiles of the action gap. A plot of a neonatal arm movement where *k*_*Y,G*_ = 0.33 is given in Figure 2. The higher the value of *k*_*Y,G*_ (for 0 <*k*_*Y,G*_ <1), the more delayed is the peak velocity, the shorter and steeper is the final deceleration, and so the action gap closes with more “oomph” (Lee et al., 1999; Delafield‐Butt, Galler, Schögler, & Lee, 2010).

## 2 MATERIALS AND METHODS

### 2.1 Experimental set‐up

Data were obtained from 10 healthy newborn infants (“full‐term”, 24–72 hours old, six female and four four male) who were in‐patients with their mothers for postpartum care in the Postnatal Wards at the Simpson Centre for Reproductive Health at the Royal Infirmary of Edinburgh, Scotland, UK. Parents of the infants were resident within the Edinburgh area and were representative of the Scottish city's demographic. All term‐born babies (mean gestational age at birth 40 weeks 2 days, *SD* 7.7 days) were paediatrically assessed as healthy with no signs of neurological or other complication. The babies were in hospital awaiting maternal recovery from Caesarean section or minor childbirth complication. All babies were discharged shortly after recording or on the day of recording. Data obtained from a second group of eight prematurely‐born infants were deemed “at‐risk” for neurodevelopmental disorder due to their severe premature birth and low birthweight (“premie”, born < 29 weeks gestational age typically with body mass < 1200 grams, three female and five male), and were collected within one week of discharge from the hospital at near corrected term when the infants’ health was considered stable by paediatric assessment (mean corrected gestational age at discharge 38 weeks, *SD* 2 weeks 4 days). Thus, the “premie” infants were on average 2 weeks younger than the “full‐term” infants and in stable health, but at‐risk for neurodevelopmental disorder. Participant information is presented in Table 1.

**Table 1:** Participant information for the full‐term and premie groups

Infant	Sex	Gestation *a*ge (GA) at *b*irth (week, day)	Weight at *b*irth (g)	Chronological *a*ge at *r*ecording (GA+postnatal age; week, day)
Neonates				
1	f	39,0	2360	39,2
2	f	40,0	3540	40,1
3	m	40,5	3600	41,3
4	m	40,0	4340	40,3
5	f	41,3	3280	41,5
6	f	41,6	4180	42,2
7	m	39,0	3520	39,3
8	f	39,2	3374	39,3
9	f	41,5	4260	42,0
10	m	41,0	3850	41,2
Mean (SD)		*40,3 (1,1)*	*3630 (584)*	*40,5 (1,1)*
Premies				
1	m	27,0	1140	41,0
2	m	27,6	1320	39,6
3	f	26,0	900	40,4
4	m	28,0	975	34,1
5	f	28,0	1130	38,5
6	f	26,5	<1200	34,0
7	m	27,0	<1200	38,0
8	m	27,0	<1200	38,0
Mean (SD)		*29,0 (0,3)*	*1093 (163)*	*38,0 (2,4)*

Mothers were approached in the wards and asked to take part in the study. They were offered an information sheet and consent form and given time to think it over. Those who agreed to participate found a convenient time when their baby was in an awake state, typically just before feeding, and were escorted to the recording studio. The studio was assembled in a room in the neonatal ward where we established a six‐camera Qualisys ProReflex 500 motion capture system (Qualisys, Sweden) together with double audio‐video (XL1 & MV901, Canon Inc., Japan) to record both the mother and the infant simultaneously for qualitative assessment of the session. The recording space consisted of a table with bedding for the infant and a chair next to it for the mother or father who was sat at the foot of their infant. The recording space was shielded with padded 1.8 m high partition walls and softened using drapes over the camera tripods to afford the mothers some sense of intimacy and protection. We gave the parent minimal instruction and asked only that they sit with their baby as they felt comfortable, and to interact with him or her as he or she saw fit. The room was illuminated with dim ambient light typical of a neonatal intensive care ward so that the parent and features of the room were visible. Across all infants, parental interaction was quiet and supportive rather than arousing and intrusive, enabling infants to maintain a passive alert state throughout the recording.

### 2.2 Data acquisition

Before recordings, a reflective marker was attached to each wrist of the infant (Figure 1) and for some recordings another was attached to the clothing above the xiphoid process of the sternum, using elastic wrist‐bands and double‐sided sticky tape, respectively. Clothing was arranged not to interfere with the wrist‐band and xiphoid markers. The mother and baby were then left to settle themselves on the chair and table, and recording began. Up to four 5‐minute Qualisys records were obtained for each baby at frame‐capture rate of 500 per second maintaining a resolved spatial accuracy of less than 1 mm for each marker. Audio‐video was left running continuously during the session. Synchronizations between the audio‐video data and the Qualisys motion capture records were made using a bespoke trigger that initiated the Qualisys motion capture and simultaneously illuminated a small light‐emitting diode positioned on the corner of the table in view of the video cameras.

### 2.3 Movement analysis

The y‐axis of the 3D Qualisys record was positioned parallel to the infant's vertebral axis and pointing away from her feet toward her head. The y‐axis component of movement of each wrist marker was chosen for analysis because the vertebral axis is a cardinal axis of the body, since it is aligned with gravity when the body is erect and is therefore the axis along which precise control needs to be exercised (Figure 1 and 2). First, the y‐coordinate time series of a wrist marker was smoothed with a Gaussian filter using a sigma value of 8, which yielded a cut‐off frequency of about 10 Hz. The resulting smoothed data time series, *Y*(*t*), was then numerically differentiated to yield the velocity time series, $\dot{Y}\left(t\right)$. Individual movements were selected from all of the data by identifying the peak values of $\dot{Y}\left(t\right)$ in the time series. Five percent of each peak $\dot{Y}\left(t\right)$ velocity was calculated and a search made forward and backward along the time series to find the nearest value that rose from below to above that value and that fell from above to below this value. These 5% points before and after the peak value were taken to be the start and end of the y‐component of the wrist movement. Where the velocity, $\dot{Y}\left(t\right)$, rose again to a value above the peak value identified before reaching 5% of its value, the higher peak value was taken and its 5% value used to continue the search. The full breadth of movement from a rising $\dot{Y}\left(t\right)$ velocity above the 5% value to a falling $\dot{Y}\left(t\right)$ velocity below the 5% value identified a single “action unit”. Within this action unit, each $\dot{Y}\left(t\right)$ velocity peak with its corresponding period of acceleration followed by a period of deceleration was counted as a single “movement unit”. The bottom of the $\dot{Y}\left(t\right)$ velocity wells either side of the movement unit peak—or in the case of a first or final movement unit, the time when the $\dot{Y}\left(t\right)$ velocity exceeded 5% of the peak $\dot{Y}\left(t\right)$ velocity of the action unit—were taken to be the start and end of that movement unit.

### 2.4 τ_*G*_ *‐*coupling analysis

Each action unit was tested against the τ_*G*_ *‐*coupling model prediction (Equation 2). The procedure measured the degree to which τ_*Y*_(*t*), the tau of the gap, *Y*(*t*), to the end of the action unit, was coupled to the hypothesized intrinsic tau, τ_*G*_(*t*, *T*_*G*_), following the τ_*G*_ *‐*coupling equation, τ_*Y*_(*t*) = *k*_*Y*,*G*_τ_*G*_(*t*, *T*_*G*_) (Equation 2). The action unit time‐series, *Y*(*t*), was calculated by subtracting the value of *Y*(*t*) at the end of the action unit from each of the values in the *Y*(*t*) time series. Next, for each time point from the start to the end of the action, the τ_*Y*_(*t*) time‐series was computed using the formula, $\tau_{Y}\left(t\right)=Y\left(t\right)/\dot{Y}\left(t\right)$. The τ_*G*_(*t*, *T*_*G*_) time‐series was computed using $\tau_{G}\left(t,T_{G}\right)=\frac{1}{2}\left(t-T_{G}^{2}/t\right)$ (Equation 1), where *T*_*G*_ is the time interval between the start and end of the action. A recursive linear regression algorithm was applied to derive a measure of the degree to which control of an action unit, *Y*(*t*), adhered to the τ_*G*_ ‐coupling equation. The measure, the *percent τ*_*G*_ *‐coupled*, is the highest percentage of data points, up to the end of the action unit, that fit the τ_*G*_ ‐coupling equation, $\tau_{Y}\left(t\right)=Y\left(t\right)/\dot{Y}\left(t\right)$, with less than 5% of the variance unaccounted for (i.e., with *r* ^*2*^ of the linear regression greater than 0.95).^1^ The slope of the linear regression computed by the algorithm estimates the coupling constant, *k*_*Y*,*G*_, in the τG ‐coupling equation.

### 2.5 Selection of action units

All movement data were analysed and minimum thresholds applied for action unit selection. Action units with a distance of travel in the y‐direction less than 10 mm, or of duration less than 200 ms, were excluded from analysis. One hundred action units were selected from each infant by taking from the left and right hands the first 25 moving up toward the head, postero‐anteriorly, and the first 25 moving down toward the feet, antero‐posteriorly. Outliers that were two standard deviations or more above or below the mean distance of the action unit, or the mean duration, or the mean % τ_*G*_ ‐coupled were, for each infant, removed as standard. Finally, the number of action units in each category was reduced to the first 12 for evenly distributed sets across all the classes. The resulting 48 action units for each of the 10 newborn infants (giving 480 in total) and eight prematurely born infants (giving 384 in total) were analysed.

## 3 RESULTS

**Figure 3:**
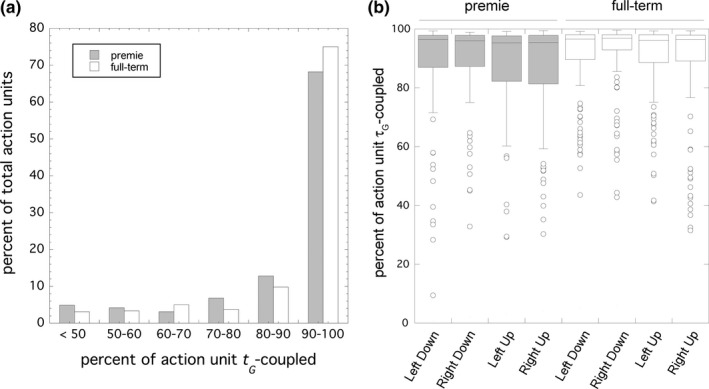
(a) The number of action units plotted against the percent of the action unit that was τ_*G*_ ‐coupled for premie (shaded bars) and full‐term (white bars). The higher the percentage, the more of the action unit conformed to the τ_*G*_ ‐guidance model, with *r* ^2^ > 0.95. Those action units where more than 90% of the movement was τ_*G*_ ‐coupled were considered to support the model. Note the reduced proportion of premie movements in this category. (b) Box plot of the percent of the action unit that was τ_*G*_ ‐coupled for each hand and each direction of movement (“up” means a postero‐anterior movement towards the head and “down” means an antero‐posterior movement towards the waist) for premie (shaded boxes) and full‐term (white boxes)

**Figure 4:**
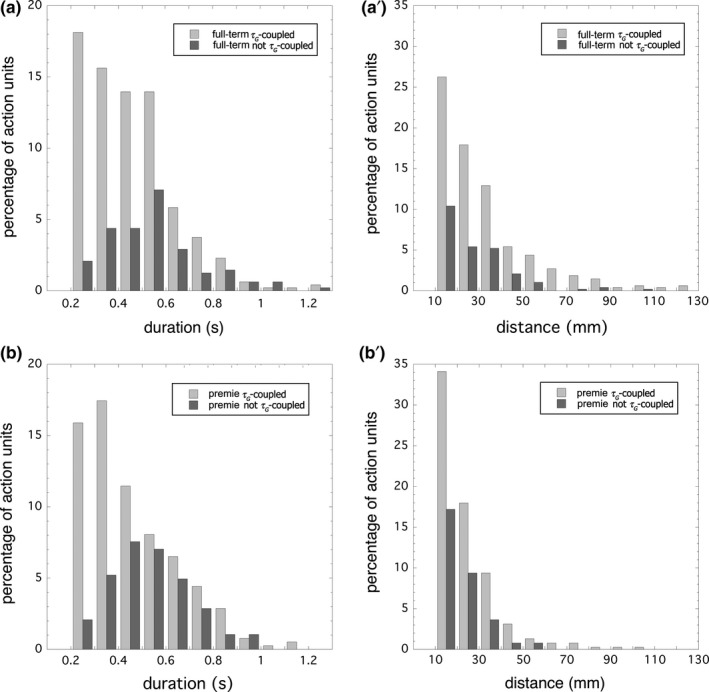
Distributions of duration and distance for full‐term (a, a’, respectively) and premie (b, b’, respectively) arm action units that conformed (*percent τ*_*G*_ *‐coupled* > 90%) and did not conform (*percent τ*_*G*_ *‐coupled* < 90%) to the τ_*G*_ ***‐***coupling model. The shapes of the conforming and non‐conforming distributions are different for duration (a, b) but not for distance (a’, b’)

**Figure 5:**
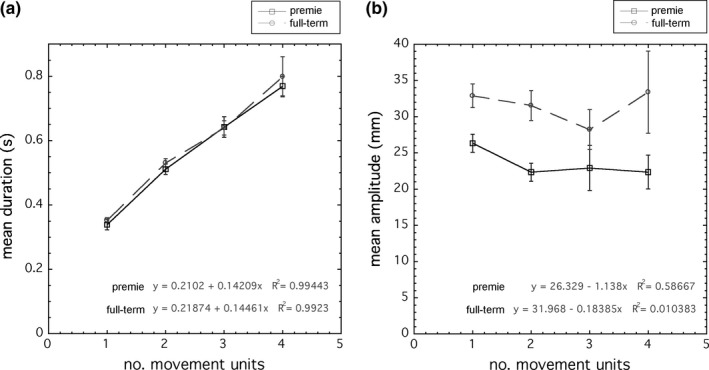
The mean (a) duration and (b) distance of action units as a function of the number of constituent movement units. Standard error bars are given. Action units increase linearly in duration with number of constituent movement units, whereas distance remains approximately constant regardless of the number of movement units. Linear regressions are shown. Note the significant reduction in premie distances, but identical durations

We identified five features of neonatal arm movements that support the tested hypothesis, and give definition to its spatiotemporal structure. In the first case, we directly tested our computational model of prospective control against data recorded from both groups of infants. We found that the majority of movements conformed to the model and therefore support the hypothesis that these movements were prospectively organized (Figure 3). Next, we compared the degree of control between healthy term‐born neonates and premies at‐risk for neurodevelopmental disorder. We found that the premies had a small, but significant, reduction in control. Further, on analysis of the motor control variables, the full‐term neonates demonstrated more structure between variables than the premies (Table 2). When controlling for age, these effects appear more pronounced, suggesting a premature birth‐related effect rather than one pertaining to chronological age. Finally, we draw out a striking feature of these infant movements. Those movements that were prospectively controlled were organized by fixed duration, but variable distance units of acceleration‐deceleration, referred to as movement units (Figure 5), a feature corroborated by comparison of full‐term and premie groups that demonstrated differences in distance, but not duration (Table 3). Further, these showed different distributions of duration and distance travelled from those that were not (Figure 4), altogether indicating a particular structure in the prospective organization of neonatal arm movements reduced in those infants born prematurely.

**Table 2:** Pearson's correlation coefficients for each of the parameters measured for each of the “τ_*G*_ *‐*coupled” action units for full‐term and premies

	Distance	Percentage τ_*G*_ ‐coupled	No. of movement units	Coupling constant, *k*_*Y*,*G*_
*Duration*				
full‐term	**0.214** a	−0.066	**0.728** a	−0.008
premie	0.035	0.026	**0.749** a	−0.111
*Distance*				
full‐term		−0.016	0.003	**0.162** a
premie		−0.009	−0.106	0.001
*Percentage τ*_*G*_ *‐ coupled*				
full‐term			0.128^b^	**−0.611** a
premie			0.120	**−0.569** a
*No. of movement units*				
full‐term				**−0.187** a
premie				**−0.212** a

a Correlation is significant at the 0.01 level.

b Correlation is significant at the 0.05 level.

**Table 3:** Means and standard deviations of the movement parameters of the τ_*G*_ *‐*coupled action units for full‐term and premies. Significances of group mean differences were tested using the *t* test

	Full‐term		Premie		
	Mean	*SD*	Mean	*SD*	*p*
*k*_*Y*,*G*_	***0.409***	***0.194***	***0.376***	***0.194***	***<.05***
No. movement units	1.631	0.847	1.755	1.022	.096
Percentage τ_*G*_ *‐*coupled	96.82	2.07	96.61	2.14	.215
Duration (s)	0.455	0.186	0.457	0.193	.939
*Distance (mm)*	***32.19***	***22.18***	***24.42***	***14.47***	***<.001***

### 3.1 Neonatal arm movements generally conformed to the τ_*G*_ *‐*coupling model

For the healthy term‐birth neonates, we found that 360 of the total of 480 action units analysed, that is, 75%, conformed to the τ_*G*_ ‐coupling model for over 90% of their movement (mean 96.8%, *SD* 2.1) with an *r* ^*2*^ greater than 0.95 (Figure 3a). These action units we considered “τ_*G*_ *‐*coupled”. A small proportion of the actions (47, i.e., 10%) exhibited τ_*G*_ *‐*coupling between 80% and 90% of their movement, and the rest of the action units (73, i.e., 15%) exhibited τ_*G*_ *‐*coupling for less than 80% of their movement. These latter action units with less than 90% of their movement τ_*G*_ *‐*coupled we considered were either not performed well or were enacted by another motor mechanism, such as an uncontrolled impulse, and hence did *not* conform to the τ_*G*_ *‐*coupling model. Taking all 480 τ_*G*_ *‐*coupled action units together, there were no significant differences between left and right arm movements (Independent‐Samples Median Test; *Grand Median* = 96.5%; *p* = .648), and up and down movements (Independent‐Samples Median Test; *Grand Median* = 96.5%; *p* = .315) in the percentage of the movement that was τ_*G*_ *‐*coupled (Figure 3b). Therefore, in subsequent analyses we focused on the 360 action units that conformed to the τ_*G*_ *‐*coupling model and considered these as a single set.

### 3.2 Reduced τ_*G*_ *‐*coupling in premie arm movements

Prematurely born infants recorded at correct term or near term showed general, but reduced, τ_*G*_ *‐*coupling in the proportion of arm movement deemed “τ_*G*_ ‐coupled”, the category of movements most efficiently controlled (Figure 3b), in comparison to healthy term‐born neonates. Altogether, 262 of the total of 384 action units analysed, that is, 68%, conformed to the τ_*G*_ ‐coupling model for over 90% of their movement (*M* = 96.6%, *SD* = 2.1%) with an *r* ^2^ greater than 0.95 (Figure 3b), a 7% reduction from the full‐term neonates. This reduction in τ_*G*_ *‐*coupling was confirmed by a Mann‐Whitney U test indicating greater overall τ_*G*_ *‐*coupling in the full‐term group (*Mdn* = 96.53) than in the premie group (*Mdn* = 95.91), *U* = 82830, *p* = .010, *r* = .087, suggesting that premies were performing less well than those born full‐term.

There were no significant differences between left and right (Independent‐Samples Median Test; *Grand Median* = 95.9%; *p* = .838), and up and down movements (Independent‐Samples Median Test; *Grand Median* = 95.9%; *p* = .307) in the percentage of the movement that was τ_*G*_ *‐*coupled across all 384 premie action units (Figure 3b).

### 3.3 Distributions of duration and distance differ between τ_*G*_ *‐*coupled and τ_*G*_ *‐*uncoupled movements

Next, we examined the distributions of the number of action units within ranges of *duration* and *distance* for the term‐birth neonates (Figure 4a) and those born prematurely (Figure 4b). We found that the distributions of the number of action units within ranges of duration differed for those that conformed or did not conform to the τ_*G*_ *‐*coupling model, but the distributions did not vary between these groups for ranges of distance. The most common range of durations for those action units that conformed to the τ_*G*_ *‐*coupling model was the shortest range, i.e., the 0.2–0.3s bin, in the case of the full‐term neonates, but the next longer, that is, the 0.3–0.4s bin, in the case of the premies. Both showed a roughly linear decrease in population in each of the ranges after, until approaching nil at about 1.0 s. There was a noteworthy level population distribution in the neonates between the 0.4–0.5s bin and the next 0.5–0.6s bin. In contrast, the commonest range of durations for those action units that did not conform to the τ_*G*_ *‐*coupling model was 0.5–0.6s for the neonates, similarly 0.4–0.6s for the premies, with an approximately bell‐shaped build and decline to either side. These data point to an appropriation of movements with durations less than 1.0 s by the τ_*G*_ *‐*coupling system, where the shorter duration movements were more strongly appropriated than the longer duration ones.

The distribution of distances was similar between those actions that conformed to the τ_*G*_ *‐*coupling model and those that did not conform, although the premies appeared shorter in distance with a more compact distribution profile—a feature evident in further statistical analysis below. Thus, from these distributions it appears that the τ_*G*_ *‐*coupling system was effecting appropriation of *movement times*, irrespective of movement distance. This phenomenon was clarified in our next finding.

### 3.4 Full‐term actions demonstrate greater structure than premie actions

We examined the 75% of action units that conformed to the τ_*G*_ *‐*coupling model for full‐term neonates, and the 68% of action units that conformed to the τ_*G*_ *‐*coupling model for premies. Because the full‐term and premie groups differed in chronological age at recording (Table 1), we controlled for age by including it as a co‐variate in partial Pearson's correlations. First, we examined duration and distance of the action units together with the τ_*G*_ *‐*coupling parameters to test for correlations between them (Table 2). A strong correlation was found between the duration of the action unit and the number of constituent movement units for both neonates, Pearson's *r*(380) = 0.726, *p* < .01, and premies, Pearson's *r*(262) = 0.748, *p* < .01. The implications of this are discussed in the following paragraphs. Further, a strong negative correlation was found between the percentage of the movement τ_*G*_ *‐*coupled and the coupling constant, *k*_*Y*,*G*_, for both neonates, Pearson's *r*(380) = −0.607, *p* < .01, and premies, Pearson's *r*(262) = 0.570, *p* < .01, showing that those movements with soft, gentle conclusions were better maintained with higher percent τ_*G*_ *‐*coupled values. In contrast, actions that concluded abruptly were not as well maintained and gave lower percent τ_G_ *‐*coupled values. Similarly, both neonates, Pearson's *r*(380) = −0.208, *p* < .01, and premies, Pearson's *r*(262) = −0.212, *p* < .01, showed a significant correlation between the number of movements units in an action and its coupling constant, *k*_*Y*,*G*_. Again, this suggests that those actions with softer, gentler conclusions were, on average, composed of more movement units. Finally, there was a correlation between the percent τ_*G*_ *‐*coupled values and the number of movement units in an action, with stronger significance in the full‐term group (Pearson's *r*(380) = .140, *p* < .01) than in the premie group (Pearson's *r*(262) = .142, *p* < .05).

It is noteworthy that the full‐term group showed more structure in the relations between motor parameters than the premies did. For example, duration was significantly correlated with distance in the case of the full‐term neonates, Pearson's *r*(380) = 0.219, *p* < .01, but not for the premies, Pearson's *r*(262) = 0.051, *p* = .414. And distance was significantly correlated to the coupling constant, *k*_*Y*,*G*_, for the full‐term infants, Pearson's *r*(380) = 0.171, *p* < .01, but not for the premies, Pearson's *r*(262) = 0.001, *p* = .992.

As an exploratory analysis in order to determine the extent to which chronological age may have influenced the difference between the premie and full‐term groups, we also tested for correlations between age and the motor control variables using a Pearson's test. Data were collapsed across groups, combining full‐term and premie data. When restricted to τ_*G*_ *‐*coupled actions, chronological age showed no correlation with the percent τ_*G*_ *‐*coupled (Pearson's *r*(622) = .076, *p* = .059) or the coupling constant (Pearson's *r*(622) = .011, *p* = .792), but did correlate with distance (Pearson's *r*(622) = .181, *p* < .000) and the number of movement units (Pearson's *r*(622) = .107, *p* = .007). When taking all data into consideration irrespective of their coupling status, chronological age correlated positively with distance (Pearson's *r*(864) = .187, *p* < .000) and % tau_G_‐coupled (Pearson's *r*(864) = .096, *p* = .005), and negatively with the number of movement units in a movement (Pearson's *r*(864) = .122, *p* < .000). Thus, an increase in age does correlate with the overall degree of τ_*G*_ *‐*coupling, but this does not account for all of the difference noted above between premie and full‐term groups. Further, an increase in age correlates with an increase in distance, and a reduction in the number of phases of acceleration‐deceleration in a movement.

### 3.5 Movement units at term are fixed duration, variable distance motor primitives

Next we examined means of the movement parameters for both the neonates and premies (Table 3). The value of the coupling constant, *k*_*Y*,*G*_, from the τ_*G*_ *‐*coupling equation, τ_*Y*_(*t*) = *k*_*Y*,*G*_ *τ*_*G*_(*t*, *T*_*G*_) (Equation 2) was calculated as the mean value of the regression slope, which estimates the coupling constant, *k*_*Y*,*G*_. *k*_*Y*,*G*_ was 0.41 (*SD* 0.19) for neonates and 0.38 (*SD* 0.19) for premies, suggesting that movements that concluded gently, as opposed to having rapid, halting finishes, were more common. There was a marginal statistical difference between groups, independent samples *t* test, *t* = −1.97, *p* = .049. On average there were 1.63 movement units (*SD* 0.85) in each of the 360 full‐term τ_*G*_ *‐*coupled action units and 1.76 movement units (*SD* 1.0) in each of the 262 premie ones, so that on average each action was composed of multiple phases of acceleration‐deceleration. The groups were statistically comparable, independent samples *t* test, *t* = 1.7, *p* > .05.

The median duration of these action units was 425 ms (*SD* 186 ms) for the neonates, and 457 ms (*SD* 193 ms) for the premies. The groups were statistically comparable, independent samples *t* test, *t* = 0.076, *p* > .05. However, the distances varied significantly between groups, independent samples *t* test, *t* = −5.283, *p* < .001, with a mean distance for full‐term neonates of 3.21 cm (*SD* 2.22 cm) larger than the mean distance for premies of 2.44 cm (*SD* 1.45 cm). The implications of this difference are brought out below.

We found a remarkably strong linear relation for both premies, *r* ^2^ = 0.99 (*F* = 356.9, *p* < .001), and full‐term neonates *r* ^2^ = 0.99 (*F* = 257.7, *p* < .001) between the mean duration of an action unit and the number of movement units in the action unit (Figure 5a). On average, an action unit comprising one movement unit was 363 ms long and as more movement units were introduced into the action unit its duration increased by 145 ms for each additional movement unit. However, there was no such linear relationship for movement distance for premies, *r* ^2^ = 0.59 (*F* = 2.839, *p* = .234), or full‐term neonates, *r* ^2^ = 0.0104 (*F* = 0.0209, *p* = .898): the distance of action units remained approximately constant, with a full‐term mean of 3.2 cm and a premie mean of 2.4 cm, regardless of how many movement units the action unit was composed of (Figure 5b). Thus, the partitioning of action units into movement units appears to be organized by regular temporal units, within which distance may vary.

## 4 DISCUSSION

### 4.1 The organization of prospective control of neonatal arm movements and its disruption by premature birth

We confirm that the movements of the neonatal arms were prospectively organized. We found that 75% of healthy, term‐birth neonatal arm movements along the vertebral axis are explained by the linear τ_*G*_ ‐coupling model, using the strict criterion that over 90% of the movement data must conform to the linear model with an *r* ^2^>0.95. This result suggests that, from birth, a predominant portion of infant arm movements made parallel to the vertebral axis are organized with a standard action pattern effected through prospective information. This coherence in the structure of infant arm movements is found equally between directions and lateralities, suggesting a generic deployment of the τ_*G*_ action system for arm motor control. However, the fact that one quarter of the movements measured were not τ_*G*_ ‐coupled suggests that the system is not fully functional at birth, or that another motor mechanism such as unguided impulses may be operative in a small proportion of infant actions.

Interestingly, those infants born prematurely and tested at corrected term or near term demonstrated reduced τ_*G*_ ‐coupling. This stands in agreement with the notion that reduced τ_*G*_ ‐coupling can indicate neurological risk or developmental delay, although this finding requires verification to test for possible confounders. In earlier work, Craig, Grealy, and Lee (2000) found reduced τ_*G*_ ‐coupling in the sucking patterns of infants with developmental delay. The prematurely born infants in our study were classified as “at risk” for neurological complication, given their premature birth and low birthweight. Thus, it appears that τ_*G*_ ‐coupling below developmental norms may provide a useful biomarker for the measure of developmental delay or neurological health, but more work is required to define these variable norms, age‐dependent effects, and thresholds of concern.

We performed some statistical controls for age‐dependent effects, because our premie group was two weeks younger on average than the full‐term group at time of measurement. These results suggest an age‐dependence where an increase in age increases the proportion of a movement τ_*G*_ ‐coupled. But the age effect does not appear to account for differences in the structured organization of movement between premie and full‐term infants.

The premie group had differences in experience in movement for some nine weeks *ex utero*, with different resistive forces and different effects of gravity from those neonates of full gestation. Less proficient generation of τ_*G*_ ‐coupled arm movements may be a function of different biomechanical constraints in conjunction with increased hypotonicity of the neck muscles due to clinical neonatal intensive care preference for the prone position during early postnatal life in clinical care. The prone position restricts opportunity to perform arm movements in the axis observed in this study, where the infants are supine. In contrast, the full‐term infants had many opportunities to perform these movements, even in the last part of their foetal life. Such differences in the kinds of actions afforded may limit premature‐born infant movement, causing the differences in control we observed. Further, some of the premies in our group may have been developmentally delayed, exacerbating any developmental differences in movement.

The small at‐risk sample size of infants with differing gestational ages presents a complex picture with regard to the exact nature of this motor disturbance. Possible confounders require examination in future work. Nevertheless, our findings demonstrate significant disruption to prospective motor control in these infants, and deliver some significant promise for future development of a computational clinical motor assessment.

Premies demonstrated reduced coupling between distance and duration, and distance and coupling constant, *k*. These particular features of control suggest a reduced structure in the organization of movement compared to term‐birth infants. Premie movements were significantly shorter in amplitude. This was likely due to smaller body size and arm length, and therefore reduced absolute distances of travel. Or it may reflect a smaller degree of action space.

Despite an extended period *ex utero* with which to develop motor experience under normal forces and gravity, motor organization appears reduced overall across the premie group. Future work is needed to test whether this reduction in control and structure was due to the age difference, differences in developmental progress, or was indicative of neurological distress. Nevertheless, although our data demonstrate reduced τ_*G*_‐coupling and reduced structure in premies in comparison to their term‐birth counterparts, the majority (68%) of the arm movements of prematurely‐born infants were also τ_*G*_‐coupled, indicating a foundation of intact prospective guidance from which development may progress. By the time an infant becomes a skilled adult, this tau coupling will have developed to be regular and very precise (Lee et al., 2009); adult sportspersons are exemplary at precise prospective control (Craig et al., 2000).

### 4.2 Invariant temporal units of movement

Interestingly, analyses of the τ_*G*_‐coupled data show that the prospective organization of movement was arranged in temporal segments of consistent durations that integrate additively, apparently irrespective of distance. This result was unexpected, because one might assume that the more phases of acceleration‐deceleration there are within an action unit, the larger that distance of travel will be. It may be that additional distances of travel occurred in the axes that were not measured, that is, the x‐ or z‐ axes, and this is a limitation of our paradigm. However, the finding of a regular pattern of movement unit durations remains accurate, despite the single‐axis measure employed in this study, and points to a fundamental temporal organization of infant arm movement. Moreover, this regular temporal structure appears impervious to developmental age or compromise: the temporal structures of movement units were exactly comparable between the premie infants and their healthy term‐birth counterparts, despite reduced distances and efficiency of control. Temporal structure, not spatial structure, appears to be a fundamental invariant.

In these data, the temporal structure appears to be formed by a base duration of 218 ms atop which movement units of 145 ms are added, so that an action unit with one movement unit is 363 ms, with two movements units is 508 ms, and so on. It is noteworthy that the 145 ms duration of the additional movement units is comparable to an adult's “online” visuomotor delay of up to 135 ms when guiding the arm during an interception task (Lee, Young, Reddish, Lough, & Clayton, 1983). It appears that the infant may be correcting her movement “online” over the course of the movement. Each time she does so, there appear velocity wells demarcating the boundaries of movement units. It further shows that, while these action units remain τ_*G*_‐coupled, they are not performed with the same degree of smoothness found in skilled adult movements. Nevertheless, the multiple movement unit composition of infant arm movements first noted by von Hofsten and Rönnqvist (1993) demonstrates an intrinsic organization within each composite action, despite these multiple acceleration‐deceleration phases.

Adult motor skills are known to improve by reducing the number of movement units in an action unit to one, which results in a smooth efficient action (Lee et al., 1999). Studies have shown that in infancy arm motor skill improves over the first eight months of life from reaches to seen objects with multiple movement units—comparable to what we observe here—to reaches with only two movement units, the latter comparable to skilled adult reaches with a minor final adjustment (von Hofsten, 1991). A central task in perceptuomotor control development is the successful integration of movement units into efficient and smooth action units.

Our finding that movement units are temporally integrated into action units supports the τ_*G*_‐coupling model, which is based on time‐to‐gap‐closure measures rather than spatial measures. The general thesis reasons that temporal (and directional) measures are the essential basic information for controlling movement (Lee et al., 2009). Direct perceptuomotor information is required to do so efficiently. Time‐to‐gap‐closure measures constitute such temporal information and can be readily coupled onto, and therefore “guide”, the muscular movements of the arm. Alternative explanations for goal‐directed motor control provided by dynamical systems equations do not have perception as an integral component (Shenoy, Sahani, & Churchland, 2013).

It appears in these data that the basic movement unit may be the basic movement primitive with which longer duration, multiple movement unit actions are built. The τ_*G*_ *‐*coupling system appears to operate to control the activity of both the basic building block and to consolidate multiple building blocks to form single, coherent whole actions. A motor control hierarchy is produced where a super‐ordinate τ_*G*_ *‐*coupling system appropriates into it the activity of the sub‐ordinate movement units. This control structure yields an umbrella of organization where movement units are subsumed under action units. This notion concurs with the neuromotor control hierarchy put forward by Powers (1973) and more recently developed into Perceptual Control Theory (Mansell & Carey, 2009). The τ_*G*_ *‐*coupling system acts as an integrative one functioning to maintain a parsimonious constancy of form of a perception‐action event.

Interestingly, our results may shed light on data on the development of “babbling hands” of infants (Petitto, Holowka, Sergio, & Ostry, 2001). Using a similar motion capture methodology to ours, it was found that the commonest movement unit duration for infants aged between 6 and 12 months was 280 ms, which is comparable to our commonest occurrence of τ_*G*_ *‐*coupled movements at birth. In contrast, sign‐language‐exposed infants exhibited an additional, stronger peak generated at 800 ms and a lessening of the peak at 280 ms. These data suggest that linguistic gestural expressions produced by babbling on the hands shifted the course of action of their hand movements to longer‐duration units. In light of our data, it appears that the purposive nature of the babble requires the integration of multiple movement unit primitives, and it is likely that the developing infant does this using the τ_*G*_ *‐*coupling system, as she is beginning to do at birth. This would also suggest that the expressive capacity of the infant develops with her motor control capacity. As she is able to integrate more complexity under single action units, so her mind is able to integrate and express greater complexity. Her developing perceptual awareness increases hand‐in‐hand with her developing perceptuo‐motor capacity and activity. This is also a central notion of perceptual control theory and agrees with the notion that consciousness rests on perceptuomotor capacity (Hurley, 1998; Clark, 1999; Noë, 2004).

This point is salient in light of the recent discovery of a possible ontogenetically primary movement disruption in children with autism spectrum disorder (Anzeluwicz, Sobota, & Delafield‐Butt, 2016; Fournier, Hass, Naik, Lodha, & Cauraugh, 2010; Teitelbaum, Teitelbaum, Nye, Fryman, & Maurer, 1998; Torres et al., 2013). Sensorimotor timing and integration of single action units appears to be fundamentally disrupted in children with autism spectrum disorder, which some authors have posited can explain consequential difficulties of engagement and learning by thwarting a basic motor intention and leading to frustration, distress and social withdrawal (Cook, 2016; Trevarthen & Delafield‐Butt, 2013; Whyatt & Craig, 2013). Precise measurement of infant motor control may one day lead to advanced early screening of neurodevelopmental disorders such as autism, but much more research is needed to characterize these movements across a population at birth, with neurodevelopmental follow‐up. In pragmatic clinical practice, the motor system may be neglected unless motor problems are severe. Thus, clinical practice today may miss important markers of risk for neurodevelopmental disorder or neuropathology. With more work in this field, improved knowledge and awareness of subtle, but significant, motor concerns may lead to sensitive instrumentation to detect early signs of neuropathology and psychopathology at birth, in the spontaneous movements of the infant.

### 4.3 A primary motor intentionality

The nature of the tau variable as hinged on a future state yields organization of action into units, each with an eye to the future. This system of prospectively organized action units is comparable to the notion of a primary sensorimotor intentionality, or *intentions in action*, that are organized by their motor goal (Delafield‐Butt & Gangopadhyay, 2013; Searle, 1980, 1983). This philosophical notion agrees with Panksepp's identification—developed through comparative neurophysiological work across the vertebrates—of a primary self that expresses its agency in movement (Panksepp, 2011; Panksepp & Biven, 2012). These basic intentions in action, or intention‐actions as Searle names them, are self‐generated future‐oriented movements whose organization is determined by an anticipation of their future effect. This is a pre‐reflective, pre‐conceptual, and prospectively perceptually organized intentionality operative before the sophisticated cognitive tools of abstraction, reflection, and planning allow for a conceptually backed, reflective intentionality more typical of adult experience as *intentions to act* (Pezzulo & Castelfranchi, 2009; Searle, 1980, 1983; Vandekerckhove & Panksepp, 2011). The observation that a majority of neonatal arm movements were prospectively organized supports the notion of a pre‐reflective, pre‐conceptual intentionality operative in neonatal movement. Primary sensorimotor intentions, *intentions in action*, appear to be “built‐in” to the fabric of neuromotor information from birth.

In our model and in the movements under consideration here, this future effect is not dependent on external sensory consequences, but rather on body space goals. It organizes the form of action in the body's action space. Myowa‐Yamakoshi and Takeshita (2006) suggest that by late foetal life a rudimentary body map is established that involves knowledge about the inter‐sensorimotor relations of the body. Our model supports the notion that movement at this early age is guided by the infant along body space‐time goals. Its organization is not necessarily coupled to external objects in the environment, such as in pre‐reaching with a specific functional task (von Hofsten, 1984). It is therefore self‐referenced and may be uniquely positioned to convey information about the particular affective experience of the infant within the form of the action pattern, what Stern calls vitality affects (Stern, 2010), precisely because it is not instrumental (seeking a functional result in object manipulation). Rather, in early life these movements are predominantly communicative, forming the basis of sharing intentions, arousal, and interest with other persons (De Jaegher, Perakyla, & Stevanovic, 2016; Delafield‐Butt & Trevarthen, 2015; Gallagher, 2008; Trevarthen & Delafield‐Butt, 2015). It will become interesting in future work to measure and define differences in form of movement (action pattern) during different social and affective conditions to better understand the possible expressive content of subsecond adjustment to these movements (cf. Rochat et al., 2013; Schögler et al., 2008; Stern, 2010).

Recognition of a pre‐reflective, pre‐conceptual primary intentionality operative at birth is an important concern for both psychological theory and clinical practice because the spontaneous arm movements made by neonates have traditionally been considered reflexes devoid of psychological qualities. The unwitting consequence of this view is that they are not expressive of feeling or intention, the basic subjective constituents of an individual self. Our observation that neonatal arm movements are a basic, primary form of intended action has important bearing on understanding the development of agency and intentionality. It stands in agreement with a growing philosophical psychological account that basic, embodied intentions are part‐and‐parcel of the core sense of self (Alcaro, Carta, & Panksepp, 2017; Delafield‐Butt & Gangopadhyay, 2013; Feinberg & Mallatt, 2016; Fuchs & Koch, 2014; Gallagher, 2000, 2005; Hohwy, 2007; Merker, 2007; Northoff & Panksepp, 2008; Pacherie, 2008; Stern, 2010; Trevarthen & Delafield‐Butt, 2017; Zahavi, 2005, 2006). This non‐verbal modality of expressive gesture made in communication with a caring and attentive other underpins preverbal, embodied intersubjective communication important for psychological development and health (Delafield‐Butt & Trevarthen, 2015; Di Paolo & De Jaegher, 2015; Rochat & Gallese, 2016; Trevarthen et al., 2015) and, in cases where the infant's mental health or development is threatened, may require professional assistance to support (Brazelton, 2006).

## 5 CONCLUSIONS

In sum, 75% of the healthy full‐term neonates’ and 68% of prematurely‐born infants’ arm movements analysed appear to have been controlled over 90% or more of the movement using a prospective *τ*_*G*_ *‐*guidance system that coupled the physical movement of the arm onto an internal τ_*G*_ action pattern. We conclude that this system enables a primary sensorimotor intentionality operative at birth in the control of movement. Further, its prospective motor structure appears to be organized by regular temporal, but variable, spatial units. In infants born prematurely and considered “at‐risk” for neurodevelopmental disorder, the efficiency of prospective control was reduced, indicating a disruption to efficient self‐generated action that may have downstream developmental consequences for neuropsychological health, with implications for care. Altogether, these data show that neonatal arm movements are prospectively temporally organized, display reduced organization and control when under neurological stress, and are organized by regular temporal units irrespective of distance. Prospective control appears a fundamental structure in movement from birth, giving a strong embodied foundation to the development of human intentionality, empowering agency.

## ENDNOTE

1 The data points excluded by the recursive regression algorithm were those at the start of the movement, which therefore had the highest *τ*_*Y*_(*t*) values, and so were most likely subject to measurement error.
